# Characterization of statistical features for plant microRNA prediction

**DOI:** 10.1186/1471-2164-12-108

**Published:** 2011-02-16

**Authors:** Vivek Thakur, Samart Wanchana, Mercedes Xu, Richard Bruskiewich, William Paul Quick, Axel Mosig, Xin-Guang Zhu

**Affiliations:** 1 Chinese Academy of Sciences and Max Planck Society (CAS-MPG) Partner Institute for Computational Biology, Key Laboratory of Computational Biology, Shanghai Institutes for Biological Sciences, Chinese Academy of Sciences, 320 Yueyang Road, Shanghai 200031, PR China; 2 International Rice Research Institute (IRRI), DAPO Box 7777, Metro Manila, Philippines

## Abstract

**Background:**

Several tools are available to identify miRNAs from deep-sequencing data, however, only a few of them, like miRDeep, can identify novel miRNAs and are also available as a standalone application. Given the difference between plant and animal miRNAs, particularly in terms of distribution of hairpin length and the nature of complementarity with its duplex partner (or miRNA star), the underlying (statistical) features of miRDeep and other tools, using similar features, are likely to get affected.

**Results:**

The potential effects on features, such as minimum free energy, stability of secondary structures, excision length, etc., were examined, and the parameters of those displaying sizable changes were estimated for plant specific miRNAs. We found most of these features acquired a new set of values or distributions for plant specific miRNAs. While the length of conserved positions (nucleus) in mature miRNAs were relatively longer in plants, the difference in distribution of minimum free energy, between real and background hairpins, was marginal. However, the choice of source (species) of background sequences was found to affect both the minimum free energy and miRNA hairpin stability. The new parameters were tested on an Illumina dataset from maize seedlings, and the results were compared with those obtained using default parameters. The newly parameterized model was found to have much improved specificity and sensitivity over its default counterpart.

**Conclusions:**

In summary, the present study reports behavior of few general and tool-specific statistical features for improving the prediction accuracy of plant miRNAs from deep-sequencing data.

## Background

MicroRNAs (miRNAs) are ~21 nucleotides long sequences, which are endogenously generated both in plants and animals. They are one of the key players in gene regulation, typically inhibitory in nature, and act either at the post-transcriptional level (by triggering target messenger RNA degradation) or at the translational level (by inhibition of translation) (see review by [1]). In plants, miRNA genes are initially transcribed as a primary miRNA sequence (i.e., pri-miRNA), which folds into a hairpin loop with small overhanging regions at both ends. The pri-miRNAs are then processed into hairpin sequences (i.e., precursor miRNA) by a riboendonuclease, named DICER. The DICER protein removes the loop region from the hairpin, and the remaining duplex is transported out of the nucleus, where the complementary sequence (also called star) is removed to allow mature miRNA to be ready for action [2].

Various tools have been developed to discover conserved and/or novel miRNAs. Initially, the miRNAs have been discovered by direct cloning and sequencing [3,4], which suggested a high degree of sequence conservation of miRNA across species [5]. With the availability of a complete genome sequence, it is possible to use computational approaches to discover the miRNA homologs. Recent progress in high-throughput sequencing further enabled genome-wide discovery of miRNAs, including novel miRNAs, and their expression profiling.

Over the past few years, several web-servers and standalone applications, analyzing deep sequencing data for miRNA discovery and/or expression profiling, have been reported; the web-servers include miRCat [6], miRAnalyzer [7], miRTools [8], whereas the standalone tools include miRDeep [9], miRExpress [10], MiroPipeline [11], etc. In addition to the above categories, there have been few miRNA discovery studies, reported in species, like *Arabidopsis *[12] rice [13], maize [14], and human [15], largely carried out using in-house scripts. There are two major limitations with these tools or protocols: 1) several of them use known miRNAs to identify only the homologs present in deep-sequencing data, thus limiting the scope of discovery of *novel *miRNAs, despite the availability of complete genome sequence; 2) several others are available as web-servers, which constrains the analysis in terms of upload/analysis time for larger datasets and also by limitations in the choice of reference genomes. In contrast, miRDeep does not have these constraints. In addition, miRDeep scores the predictions, which makes it easier to pick the better candidates from the rest [9].

The scoring used in miRDeep is based on computation of posterior probabilities of statistical features, like minimum free energy (MFE), conservation of the core region of mature miRNA, etc.. Some of these features are commonly used by other tools/analyses (see Methods for details). The posterior probabilities are usually computed based on parameters derived from a known set of real and background precursors; the current parameter set used in miRDeep is from a nematode, *C. elegans*, and reported to be effective for other animals as well. However, there are major differences between the properties of plant and animal miRNAs. For example, the maximum length of plant miRNA precursors can be about ~900 nt long; the extent of pairing and bulge size of duplex (of mature and star) in plants is very different from that of animals [2]. Therefore, the differences in properties between plant and animal miRNAs can affect the parameters required for statistical scoring used in miRDeep.

In the current study we examined the key differences between plant and animal miRNAs, and their effects on general and miRDeep-specific statistical features, and further estimated the plant specific parameters of these features. With these new miRDeep parameters, we validated the prediction by applying it to a set of newly discovered miRNA in maize seedlings. The results were compared to that obtained using default miRDeep parameters.

## Results

### Frequency distribution of Minimum Free Energies of plant miRNA precursors

**Figure 1:**
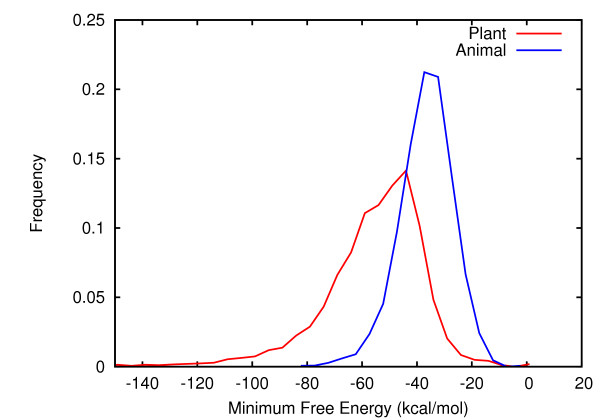
**Frequency distribution of Minimum Free Energy of all known miRNA precursors of plants and animals**.

**Figure 2:**
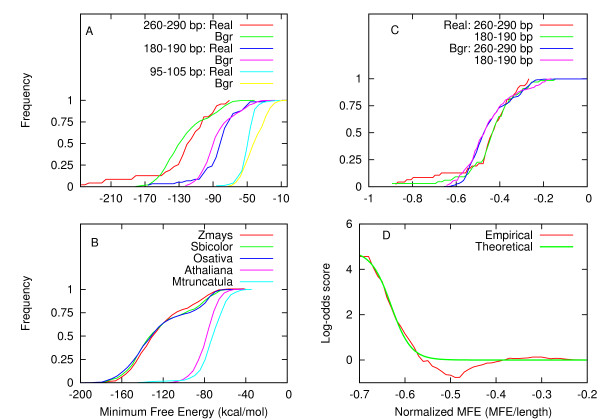
**Behavior of Minimum Free Energy distribution in plants**. (A) Cumulative distribution of MFE of real and background precursors of three different lengths: 95-105 nt, 180-190 nt, and 260-270 nt. The Bgr indicates background precursors (from maize genome). (B) Cumulative distribution of MFE of background precursor of size 260-90 nt from five plant species. The source species does affect the nature of distribution, and they tend to cluster into monocots and dicots. While the differences in distributions within the group were insignificant (at confidence level <0.05), those from two groups were highly significant. (C) Same as in A, but the MFE has been normalized by its length. For clarity the distribution of only two values of length have been shown. (D) Empirical distribution of log-odds score of MFE and its best fit theoretical approximation by a modified sigmoid function. The probabilities for background precursors, used in computing log-odds, were average of that of three monocots, namely *Z. mays*, *S. bicolor*, and *O. sativa*.

The frequency distribution of minimum free energies (MFE or *abs*) of real plant and animal miRNA precursors showed large differences: plant miRNA precursors showed a broader distribution and a lower mean MFE compared to that of animals (Figure 1). An underlying factor likely to be accountable for this difference is the length of precursors. While the length of animal miRNA precursors mostly lie in the range of 45-215 nt (mean = ~87 nt), those from plants show large heterogeneity, lying in the range of 55-930 nt (mean = ~146 nt). An examination of a relation between precursor length and MFE showed a linear relation between them, with the standard deviation of MFE, interestingly, increasing with length (Additional file 1: Figure 1). The effect of length on MFE was also evident when the cumulative frequency distribution (instead of mean) of MFE was plotted (Figure 2A). The distributions were distinct for precursors of different length. When these distributions were compared with that of background precursors of corresponding length (maize genome has been the source of background precursors unless specified), they were largely overlapping, except in the left tail region (Figure 2A). This indicated that the minimum free energy may not be a strong discriminant between real and background precursors of plants.

In order to examine if the source (species) of precursor sequences has any bias on the above observation, the MFE distribution of real and background precursors from four additional plant species (with complete genome sequence), namely sorghum (*S. bicolor*), rice (*O. sativa*), *Arabidopsis thaliana*, and *Medicago trancatula*, were compared. While the MFE distributions of background precursors of sorghum and rice converged to that of maize, the distributions of *Arabidopsis *and *Medicago *were very distinct (Figure 2B). This led us to speculate that differences possibly exists at the level of monocots and dicots. For real precursors, although differences did exist between distributions of monocot and dicot species, however, these were statistically insignificant (at confidence level ≤0.05) (data not shown). These observations imply that the miRDeep training, in a plant species, is largely independent of the choice for the real precursors, but sensitive for the background precursors. This also affects the log-odds of MFE in dicots, as the MFE distributions show large differences between real and background (Additional file 2: Figure 1), as against the monocots (Figure 2A).

The multiple distributions, characterizing MFE of plant miRNA precursors (Figure 2A), posed practical difficulties in employing them in a miRNA prediction algorithm. Given the linear dependency between the MFE and the length of precursors, normalization of MFE by the length proved a better solution to the problem of multiple distributions. As shown in Figure 2C, the cumulative frequency distributions, of precursors of varying length, overlapped almost completely. For theoretical approximation of these distributions, 'Gumbel' or 'Extreme-value' distribution functions were the ideal choice, as the MFE values are 'minimum' over all possible free energies. On fitting these functions, the sum of errors, for the best fit curve, was within the allowable limit of 10% (Additional file 3: Figure 1). However, the quality of fit with empirical distributions in the left tail region was poor (in Additional file 3: Figure 1, note the region of the plot for normalized MFE values ranging between -0.4 to -0.1). A better approximation of left tail regions is desirable, as it has the potential to contribute larger scores. As an alternative the log-odds scores were directly computed from the two distributions, and were approximated by a modified sigmoid function, which fitted the left tail of curve very well (Figure 2D). The new parameters are listed in Table 1.

**Table 1:** List of miRDeep parameters estimated for plant specific miRNAs.

Feature		Original parameters of miRDeep	Plant specific (monocot)	Dicot specific (if any)
**MFE**	Known	Cumulative Distribution Function: $F(x)=e^{-e^{(x-location)/scale}}$ Location = 32; Scale = 5.5	Log-odds score: $f(x)=\frac{a}{\left(b+e^{x*c}\right)}$	Log-odds score:$f(x)=\frac{a}{\left(b+e^{x*c}\right)}$
			
	Background	Cumulative Distribution Function: $F(x)=e^{-e^{(x-location)/scale}}$ Location = 23; Scale = 4.8	a = 1.339e-12 b = 2.778e-13 c = 45.843	a = 4.46e-4 b = 9.125e-5 c = 26.929

**Stability (log-odds)**	Stable	1.6	1.37	0.63
	
	Unstable	-2.2	-3.624	-3.17

**Nucleus conservation (log-odds)**	Conserved	3	7.63	
	
	Non-conserved	-0.6	-1.17	

**Excision length**		140 nt	300 nt	

**Paired**	Total	≥14	≥15 nt	

**Unpaired**	Total	NA	≤5 nt	
	
	Consecutive unpaired	NA	≤3 nt	

**Bulge**	Total	could be as high as 5 nt ^#^	≤2 nt	

**Maximum multiple hits of deep-seq read**		5	20	

The cumulative distribution of MFE of known and background precursors, in miRDeep algorithm, are represented by Gumbel distribution functions, where *F(x) *is the cumulative frequency of MFE less than or equal to variable *x*, and *location *is used for mean centering the distribution of MFE. On the other hand, in the parameterized algorithm, log-odds of MFE were directly obtained from the distributions of real and background precursors, and then represented by a modified sigmoid function where *f(x) *denotes log-odds of a given normalized MFE. ^#^The size of bulge was estimated based on allowed difference between length of mature and star sequences in miRDeep. The bulges often appear when one of the duplex sequences has additional bases than its counterpart.

### Stability of secondary structure of plant miRNA precursors

Another feature which distinguishes real precursors from background precursors is the marked difference in secondary structure when the sequence of a candidate precursor is shuffled (referred henceforth as stability). Bonnet *et al*. [16] reported that MFE of real miRNA precursors significantly differ from their shuffled counterparts. The miRDeep implements this feature through the use of a tool RANDfold [16] to distinguish real and background precursors by examining the stability of secondary structure on shuffling. RANDfold first generates an ensemble of shuffled sequences, either by mononucleotide or dinucleotide shuffling, and computes the frequency (*p-value*) of shuffled sequences exceeding MFE of the candidates in question. So a *p-value *of 0.01, for instance, signifies that only 1% of the shuffled sequences have similar or better folding than the candidates. Typically, those candidates with a *p-*value of up to 0.05 are categorized as *stable*.

**Figure 3:**
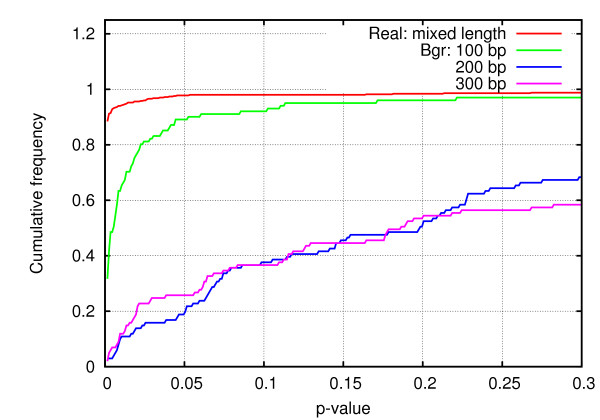
**Cumulative frequency of *p-value *of real and background miRNA precursors**. Three different lengths were examined: 100, 200, and 300 nt. At a threshold of ≤ 0.05, there was small difference in frequencies for real and background of length 100 nt. However, with increase in length of background precursors, the p-value declined drastically.

For the real precursors, the RANDfold was run on a sample of 500 precursors, sampled across all lengths, which yielded ~0.98 of them as stable (Figure 3). Like the MFE distribution, the length of miRNA precursor may also affect the stability of structures. In order to know how longer precursors behave, this program was executed on precursors with length ≥ 400 nt. There was a slight, but statistically insignificant, decline in the frequency of stable precursors (data not shown). On the contrary, the frequency of stable background precursors differed substantially for varying length. RANDfold was run on background precursors of three distinct lengths- 100, 200, and 300 nt. As Figure 3 demonstrates, the frequency of stable precursors varied considerably: the frequency for 100 nt long background precursors was slightly lower (0.8), the same declined drastically for longer background precursors (ranging from 0.18-0.25). Considering the excision length (details in later section), the frequency for the ~300 nt long background precursors was chosen as the parameter for statistical scoring (Table 1).

We further examined whether the choice of source of background precursors affected the stability. As in the case of MFE, the stability of the precursors differs substantially between monocots and dicots (Additional file 4: Figure 1). The dicot background precursors were found to have a higher fraction of stable structures (for example, 50-55% for 300 nt size range), as opposed to their monocot counterparts (only 20-25% for the corresponding length). Therefore, in dicots, the higher frequency of stable background precursors will make the distinction between real and background precursors more blurred.

### Core conservation in mature miRNAs of plants

**Figure 4:**
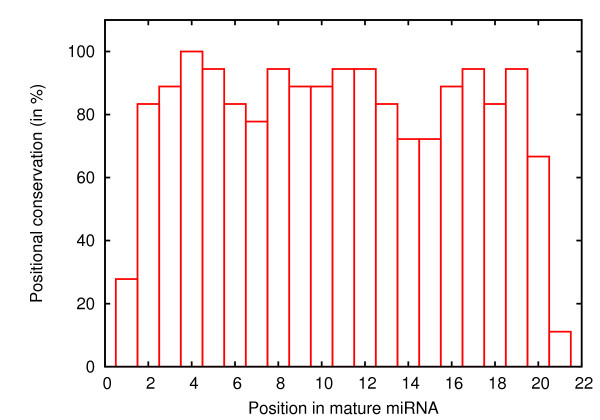
**Positional conservation in 18 plant miRNA families**. The height of a bar at any position indicates the number of families in which said position was conserved.

The mature miRNAs generally show high conservation within a family. Moreover, for a particular miRNA family, conservation is also observed across species within (plant or animal) kingdoms. Apart from nucleotides, the conservation also shows positional pattern, that is, certain positions within mature miRNAs are consistently conserved even across miRNA families. This may have some distant implications on the nature of binding of miRNA to its target, as it is also position specific. In animal miRNA families, positions 2-8 are often regarded as the nucleus or core region, and the same has been implemented in miRDeep algorithm. However, given the better complementarity between mature and star sequence in plants [2], we examined the occurrence of any deviation in the positional conservation profile. A dataset comprising 18 plant miRNA families were randomly sampled, such that each family had representatives from at least four species. Results showed that the nucleus conservation pattern in plants is quite different from that in animals (Figure 4). Considering 75% as the threshold for positional conservation across species, two distinct conservation blocks were identified: 2-13 and 16-19. For parameter estimation, the frequency of conservation at position 2-12 was found adequate; this choice was based on simulations (data not shown) for finding the minimum length of nucleus at which specificity of match of any miRNA within its family is maximized. To compute the frequency of conservation among real miRNAs, the complete set of real miRNAs were split into two sets, 10% was used as a test set and its conservation at specified positions was checked in the remaining 90%. This was repeated 10 times with disjointed test-sets. For background miRNAs, conservation was examined in a complete set of real miRNAs. While the frequency of conservation in real miRNA was 0.69, those for the background was based on a pseudo-count of 1/3000 (= 0.0003) (see Table 1 for estimated parameters).

### Precursor excision length

**Figure 5:**
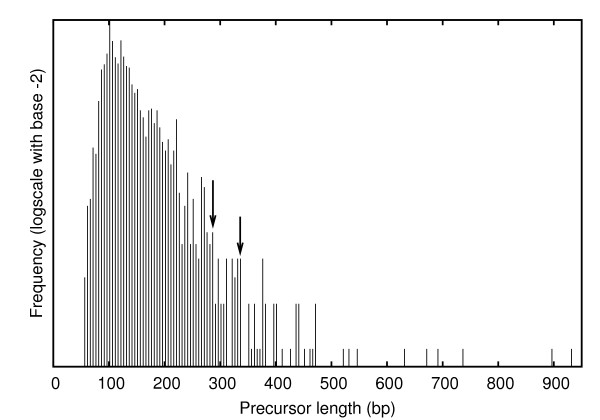
**Frequency distribution (density and cumulative) of precursor length**. Two of the possible thresholds which cover 96% and 98% of the real precursors have been indicated by down-arrows (↓). At these lengths, a reasonable sample size of miRNAs were available for estimation of parameters.

Several miRNA discovery tools, whether based on purely computational approaches or deep sequencing data analysis, involve excision of genomic regions based on the occurrence of an inverted repeat or mapping of sequencing reads, respectively. These excised precursors are subjected to secondary structure prediction to check for imperfect fold-back or hairpin structures. Since the length of plant miRNA precursors varies widely, from 50 to 900 nt (Figure 5), an appropriate choice of excision length becomes necessary. It will be inappropriate to use the maximal length as excision length, because too few real precursors of that length would be available to estimate the parameters. Moreover, longer precursors also tend to have large variations in MFE, which is likely to decrease the prediction accuracy (Additional file 1: Figure 1). Therefore, an optimal choice would ensure 1) the majority of plant precursors get covered, and 2) adequate sample size of real precursors (greater than 30) is available for parameter estimation. Two of the optimal lengths, 277 and 336, which cover 96 and 98 percent of sequences, respectively, were found to agree with the above stated criteria (Table 1).

### Other miscellaneous properties

Unlike animal miRNAs, plant miRNAs show better complementarity with miRNA star. Based on the hairpins available in miRBase release-14, the miRNA-miRNA* duplexes were found to have a maximum of 5 unpaired bases (however the majority have up to 3 mismatches), two consecutive unpaired bases, and bulge size up to 1 (Table 1 and 2). For an accurate prediction of plant specific miRNAs by any tool including miRDeep, these properties must be taken into account.

**Table 2:** Duplex (miRNA-miRNA*) associated parameters in miRDeep compared to plants miRNAs.

	miRDeep	Plants
Total unpaired	Up to 8 (for 22nt long miRNA)	Up to 5

Consecutive unpaired	Up to 8	Only 3

Total bulge	Up to 5-6	1

Consecutive bases in bulge	Up to 5-6	Nil

While computational structure prediction yields minimum free energy structures according to the Turner model [17], the way miRNA precursors fold in their cellular context may differ from the one predicted by this model. This could be more likely for longer miRNA precursors as they may have higher degrees of freedom in which to be folded. Therefore, we examined how faithfully the software RNAfold predicted the secondary structure of real plant precursors longer than the mean length. We used RNAfold [18] to generate structures of 11 real plant miRNA precursors, of size in the range of 375-425 nt. The predicted structures were consistent with the secondary structures reported in miRBase [19].

The probability distribution of mapping of reads to the real and background precursors modeled by geometric distribution in miRDeep, was assumed to be same in plants, as the nature of mapping of reads to mature-loop and star-region are likely to be the same in both plants and animals. However, a minor difference (in the distribution) cannot be ruled out due to the longer loop region in plants.

### Validation of predictions by miRDeep parameterized for plant miRNAs

In order to validate if the above parameterization improves miRDeep's prediction accuracy for plant miRNAs, we selected recently discovered miRNAs in maize as a reference set [14]. These miRNAs were not covered by the training set (miRBase release 14) used in our study. The reference set included about 150 miRNAs at the whole genome scale, and a large fraction of them were experimentally validated. In this study, we report the results for maize chromosome 5, which was reported to have 23 miRNAs transcriptionally active in maize seedlings [14]. The parameterized miRDeep predicted 17 candidates with log-odds score well above 0, and 16 of them were found to overlap with the reference dataset (Table 3). Out of the seven known miRNAs which were missed out, discovery of six of them failed as these miRNAs were either located within coding sequences (CDS) or showed high homology to CDS, thereby, reads corresponding to such cases have been excluded from our prediction. Although, this can be considered as a minor methodological limitation that reduces sensitivity, keeping this filter ON, helps minimize the false positives originating from CDS.

**Table 3:** Validation of miRNAs identified by parameterized miRDeep.

miRNA Family	miRNA name	Confirmation by RACE	Seedling RPM	If identified by parameterized miRDeep	Reads aligned	Star (count)
miR156	zma-MIR156d		14111.2	✔	5932	1

miR156	zma-MIR156l		12645.91	✔	1659	1

miR160	zma-MIR160b	✔	292.65	miRNA overlapping with CDS		

miR162	zma-MIR162	✔	2.74	poor abundance of reads		

miR166	zma-MIR166d		314.6	✔	328	1

miR166	zma-MIR166k	✔	6.52	miRNA homologous to CDS		

miR166	zma-MIR166m	✔	49.75	miRNA homologous to CDS		

miR167	zma-MIR167b	✔	2860.25	✔	17402	1

miR167	zma-MIR167c	✔	2872.95	✔	72684	0

miR168	zma-MIR168a		10364.43	✔	32179	1

miR169	zma-MIR169f		1234.4	✔	3371	0

miR171	zma-MIR171f	✔	15483.87	✔	78	1

miR171	zma-MIR171m		4.12	✔	508	1

miR172	zma-MIR172b		134.49	✔	81	1

miR390	zma-MIR390b		2220.41	✔	6504	1

miR394	zma-MIR394a	✔	4.46	MiRNA overlapping with CDS		

miR396	zma-MIR396f		849.46	✔	2503	1

miR396	zma-MIR396g		43.23	miRNA homologous to CDS		

miR397	zma-MIR397b		556.13	✔	1628	1

miR399	zma-MIR399h		43.23	✔	129	1

miR399	zma-MIR399i		90.57	✔	272	0

miR529	zma-MIR529		359.2	✔	1050	1

miR827	zma-MIR827		711.89	miRNA overlapping with CDS		

Unclassified	?		-		334	0

The candidates were compared against a recently discovered set of miRNAs, from chromosome 5 of maize, expressed in seedlings [14]. The data in third and fourth columns are from [14], while those in last two columns are from current study. The cases missed out by parameterized miRDeep appeared mainly due to a filter which discarded reads mapped to coding sequences (CDS). Even chance homology of a miRNA, either complete or at least in mature/star region, to any CDS can result into the miRNA being unidentified. RPM: Reads Per Million.

The miRDeep algorithm with default parameters, on the contrary, predicted only two candidates from chromosome 5 at a log-odds score of >0, and both failed to match known miRNAs. On a whole genome scale, it predicted a total of sixteen candidates, out of which only two overlapped with known miRNAs. When checked for the presence of basic features typical to plant miRNAs, we found the majority of them failed to meet the desired metrics. Most of the discrepancies are related to the number of mismatches in the duplex region, the bulge size, non-specific homology, or precursor length, etc. Therefore, the newly parameterized miRDeep consistently identified known miRNAs (from chromosome 5, expressed in maize seedlings) with higher specificity and sensitivity than the original parameterization.

## Discussion

The plant miRNAs differ from animal miRNAs in several aspects, mainly in the hairpin length and in the nature of complementarity with the star sequence [2]. Although the length of mature sequences largely remains the same, the length of the loop region differs substantially in plants, owing to their recent evolution [19]. These differences strongly influence the statistical features used for their prediction.

The minimum free energy (MFE), a commonly used measure for characterizing secondary structure of different types of RNA [20,21], is also being used for characterization and/or prediction of miRNAs [3,4,22-24]. For screening miRNA candidates, the majority of previous studies have either used a fixed MFE threshold (for example, -18 kcal/mol) [13], or a variable threshold [25]. The miRDeep, however, involves comparison of (posterior probabilities of) MFE of real and background hairpins, enabling a more robust discrimination between them. This comparison in plants, however, did not prove to be so straightforward, as diversity in miRNA hairpin length results in multiple distributions of MFE.

Although, the effect of hairpin length on MFE in plants has been reported earlier [25], these reports did not give a systematic evaluation of potential impacts of this relationship on prediction of plant miRNAs. In the present study, we observed that the MFE distributions become length-free by normalizing the MFE of precursor with its length, which renders the MFE of hairpins, of different length, comparable.

We further observed only minor differences between the MFE distributions of real and background precursors of comparable length. This suggested that MFE alone may not be a good discriminator between real and background miRNAs, and more weight should be placed on other measures besides MFE. These findings however do not hold for dicot species, as they do show substantial differences between real and background, rendering MFE a more important discriminating feature.

Differences between the secondary structures of candidate precursors and their shuffled counterparts is another important feature exploited for miRNA prediction [9]. The real precursors generally display substantial difference in the nature of folding (as well as in MFE) from their shuffled counterparts [16]. This difference is quantified by the p-value, which is the fraction of shuffled sequences with MFE lower than original precursors; a candidate with *p-value *≤0.05 can be statistically considered as stable. Although, plant and animal miRNA precursors, of the same length, are expected to have a similar frequency of stable precursors, due to the diversity in length of plant miRNA precursors, the parameter estimated for animals becomes inapplicable to plants. In the latter case, while p-values of real precursors remains almost the same even with increased length, that of background precursors declined substantially with length. These criteria become more effective when it comes to predicting longer candidate precursors, which is often the case with plants.

Furthermore, the conservation of mature miRNAs is yet another important feature for miRNA discovery, exploited by miRDeep and several other tools, where the former takes into account the conservation in the nucleus region of mature miRNAs [9]. The positions which constitute the nucleus in animals are 7-8 nt in length, starting from position 2 [27-29], whereas in plants, there is near-perfect complementarity all along its length. We examined the nature of the positional conservation pattern in plants, and found a relatively longer conserved motif, wherein two conservation blocks were apparent: positions 2-13, and 16-19, with position 4 completely conserved. Implementation of positional conservation pattern in plants has improved the specificity of miRNA homolog prediction.

Since plant miRNA precursors show a relatively broader distribution of length compared to animals, this in turn, necessitates a different choice of excision length(s) for candidate prediction. While Sunkar et al. [13] considered 200 nt as a threshold at which 90% of the real precursors in rice were covered, Jones-Rhoades et al. [30] took a higher precursor length (500 nt) for prediction in *A. thalina/O. sativa*. In another study by Adai *et al. *[25], in *A. thaliana *again, the maximum precursor size was set to 400 nt. Based on the distribution of length of plant miRNA precursors from miRBase database (release 14), length(s) which covered the maximum number of miRNAs, and at the same time, had an adequate number of precursors (30, for instance, which have properties of a normally distributed population) for parameter estimation, were chosen. We observed two thresholds satisfying the above constraints, 276 and 336, covering 96% and 98% of the population, respectively.

To show how much the new parameterization improves the prediction accuracy, we used the miRDeep with the default parameters to predict miRNA candidates. Results suggested that a major fraction of miRNAs, predicted using the default parameters, did not match with experimentally identified miRNAs. The observed values of key features, such as number of total mismatches, bulges, nucleus conservation, excision length, etc., of the predicted candidates were atypical for plant miRNAs. Moreover, shorter nucleus size in default miRDeep led to identification of several false miRNA homologs. However, prediction using new parameters on the same dataset showed very high prediction accuracy, with good sensitivity and even better specificity.

Notably, any proposed improvement in plant miRNA discovery must meet the criteria laid out for miRNA annotation in plants [12,31]. Despite the parameter adjustments in the miRDeep algorithm, the primary criterion for miRNA annotation, namely precise excision of mature miRNA from the stem of a stem-loop precursor, is implemented faithfully. The parameterization doesn't interfere in miRDeep's core method. Further, two of the miRDeep's statistical features, namely characterization of stem-loop and mapping of reads onto precursors, are enough to prevent a siRNA being misclassified as miRNA. Besides, there have been recent reports of few plant miRNAs being processed by riboendonucleases other than DCL1 [32], therefore, the predictive methods should also be capable of their identification. This however does not pose much problem to the tools based on deep-sequencing reads, as their methods are guided primarily by the sequences. So, a DCL3 processed miRNA, for instance, will be analyzed just like the DCL1 generated miRNAs, despite the longer product size of the former. Furthermore, there have been rare reports of multi-functional stem-loops [12], which poses challenges to the tools available for miRNA discovery. We are skeptical about the ability of the current form of miRDeep algorithm to handle such complexity.

This study also brings forward some issues that can be studied in the future. Increasing the number of plant genomes can allow researchers to further test whether MFE distributions of monocots and dicots truly differ and if so, study the underlying mechanisms. Furthermore, improved genome annotation will also improve the discovery of miRNAs missed out due to overlap with an otherwise incorrectly annotated CDS. Other desired advancements include modules for identification of other kinds of sRNA and the ability to characterize multi-functional stem-loops.

## Methods

### Known miRNA sequences

The sequences of precursor and mature miRNA of plant and animal species were downloaded from miRBase (release 14) [19] and pooled into respective sets. Additionally, plant specific miRNA sequences were also extracted (as on April 15^th ^2010) from the 'Plant miRNA Database' [33], which contains additional numbers of precursors, which are largely computationally predicted.

This dataset was further processed to remove redundancy, as in several instances, the precursor sequences are almost identical except for a few changes in nucleotides. Such sequences are likely to create bias towards the over-represented members of a miRNA family. This bias may affect some of the important analysis such as cumulative distribution of MFE and the effect of length on MFE. For obtaining non-redundant sets, the precursors were first divided into individual families and were then subjected to multiple alignment. Based on the alignment, the highly similar sequences were manually discarded, resulting in a set of 1904 precursors out of 2034.

### Generation of background sequences

A background miRNA precursor is one that exhibits similar physical properties as that of real precursors, however, they are never transcribed into a miRNA. Since it is known that plant miRNAs are transcribed largely from the intergenic region and coding sequences are unlikely to contain any miRNA. Therefore, we used protein-coding sequences of five different species namely maize [34], sorghum, rice, *Medicago*, and *Arabidopsis *(sequences of remaining four species were obtained from [35]), for generation of background precursors (in the present study, maize was the default choice for background sequences unless otherwise specified). We used *de novo *miRNA discovery program, miRCheck [36], for identification of background sequences, over a broad length range, with default parameter settings, except the excision length was increased to 500 nt.

### Statistical scoring in miRDeep

The miRDeep score is given by,

(1) $$score=log\left(\frac{P(pre|data)}{P(bgr|data)}\right)$$

where *P (pre|data) *is posterior probability of a test precursor being a 'real precursor' (*pre*) given the values of multiple statistical features (*data*), and *P (bgr|data) *is posterior probability of a test precursor being 'background precursor' (*bgr*) given the *data*.

When Eq. (1) is expanded using Bayes theorem,

(2) $$score=log\left(\frac{P(data|pre)P(pre)}{p(data|bgr)P(bgr)}\right)$$

where *P(data|pre) *is conditional probability of observing *data *in real precursors, *P(data|bgr) *is conditional probability of observing *data *in background precursors, and *P(pre) *and *P(bgr) *are prior probabilities of real and background precursors, respectively.

The *data*, for any precursor, is described by five statistical features: 1) absolute value of minimum free energy (*abs*), 2) stability of secondary structure against randomized counterparts (*rel*), 3) signature (or pattern) of mapping of reads to the precursor (*sig*), 4) sequence conservation in the nucleus (or core) region of mature miRNA (*nuc*), and 5) presence of at least one read mapping star region (*star*) of the precursor. Therefore, the term *P(data|pre)*, for instance, can be expanded into,

(3) score(data|pre)=P(abs|pre)P(rel|pre)P(sig|pre)P(nuc|pre)P(star|pre).

Out of these five probability distributions, *P(abs|pre) *and *P(sig|pre) *are continuous distributions (takes any value within specified range), while the remaining three are discrete distributions (takes one of the values from a set). The above also applies to the probability distributions for background (bgr) data. The cumulative frequency distribution of MFE, *P(abs|pre) *or *P(abs|bgr)*, shows gumbel (or extreme value) distribution, while that of signature, *P(sig|pre) *or *P(sig|bgr)*, shows geometric distribution [9]. The stability (*rel) *takes either of the two values: 0 if frequency of unstable is more than 5%, otherwise 1. Similarly, if nucleus regions is non-conserved at the specified positions, the value of *nuc *will be 0, otherwise 1. The value of *star *will be 0, if any read fails to map with star region, otherwise 1.

The miRDeep essentially involves determination of these probability distributions, mentioned in Eq. (3), for known datasets of real (*pre*) and background *(bgr) *precursor/mature sequences. Here in current study, these distributions will be estimated for plants miRNAs.

### Frequency distribution of Minimum Free Energies

The frequency distribution of MFE, plotted in Figure 1, was obtained from secondary structure prediction (by RNAfold [18]) of all known animal and plant miRNA precursors (available in miRBase release-14). To obtain a relationship between precursor length and MFE (as in Additional file 1: Figure 1), samples of plant precursors of different lengths, with sample size 60 (however for highest length, the sample size dropped to 21), each sample being homogeneous in length with a variation of 5 nucleotides (however 10 for the samples having members less than said sample size) were obtained. The MFE was computed by RNAfold and mean MFE values were plotted against the average precursor length of each sample.

For comparison of distributions of MFE of background precursors from multiple plant species (shown in Figure 2B), two-sample Kolmogorov-Smirnov tests were conducted using 'ks.test()' function of R statistical package [37].

For theoretical approximation of distributions shown in Additional file 3: Figure 1, the empirical distributions of MFE of 47 real and 554 background precursors, of length 260-290 nt, were fit with Gumbel distribution function (minimum),

(4) $$F(x)=1-e^{-e^{\frac{x}{b}}}$$

where *b *is a scaling factor. Since the fitting errors were high so the log-odds were directly computed. The MFE log-odds score, displayed in Figure 2D, were obtained by

(5) $$score(MFE)=log\left(\frac{P(abs|pre)}{P(abs|bgr)}\right)$$

The log of ratio of conditional probabilities of MFE of real and background precursors was computed for each of the bins, and were plotted. The frequency distribution of MFE was obtained with bin-size = 0.01. The empirical distribution was modeled by a modified sigmoid function [38]:

(6) $$f(normalized\_MFE)=\frac{a}{\left(b+e^{c*normalized\_MFE}\right)}$$

where *a*, *b*, and *c *are fitting parameters.

### Core conservation in mature miRNAs

To study the positional conservation in mature miRNA families of plants, we obtained all members of 18 miRNA families, sampled randomly. It was however insured that each family must have representatives from at least four species. Members of each miRNA family were aligned using CLUSTALX [39] and a position with ≥ 0.9 conservation was marked conserved. Then positional conservation profile of all 18 families were summed. That is, count of families conserved at each of the positions, starting from 1 to 23, was obtained and plotted. Since higher evolutionary divergence is likely *across *families (than *within *family), therefore threshold for defining a position as *conserved *was further relaxed, that is, ≥ 0.75 (Figure 4).

In order to obtain the frequency of nucleus conservation in real miRNAs, a strategy of 10-fold cross-validation was applied. That is, the complete set of known mature miRNAs were first shuffled, and divided into two fractions with ratio 9:1. The smaller fraction was used as a 'test set' and its conservation (at positions 2-12) was examined in sequences of larger fraction, and the frequency of sequences from test-set hitting 'training-set' was recorded. This was repeated 10 times, and an average of all ten frequencies was obtained. For background, the conservation of a large set of putative mature sequences, against the training-set described above, were examined in a similar way.

The log-odds of observing conserved (*nuc *= 1) and non-conserved (*nuc *= 0) nucleus were $score(conserved\;nucleus)=log\left(\frac{P(nuc=1|pre)}{P(nuc=1|bgr)}\right)=7.63$ and $score(non-conserved\;nucleus)=log\left(\frac{P(nuc=0|pre)}{P(nuc=0|bgr)}\right)=1.17$, respectively (Table 1).

### Stability of secondary structures of miRNA precursors

For computing frequency of stable and unstable real precursors, analysis was done on a sample of 500 plant miRNA precursors of varying lengths. Each of them were subjected to mononucleotide shuffling for 999 times, followed by computing *p-value*,

(7) $$p-value=\frac{R}{\left(N+1\right)}$$

where R is the number of shuffled sequences with MFE greater than that of original sequence, N is the number of iterations.

For background precursors, the analysis was carried on three samples of lengths: 100, 200, and 300 nt, each sample having 100 sequences. They were subjected to 499 iterations.

The estimated log-odd scores for stable (*rel = 1*) and unstable (*rel = 0*) were given by, $score(stable)=log\left(\frac{P(rel=1|pre)}{P(rel=1|bgr)}\right)=1.37$ and $score(unstable)=log\left(\frac{P(rel=0|pre)}{P(rel=0|bgr)}\right)=3.624$, respectively (Table 1).

### Running default and parameterized miRDeep on a sample deep-sequencing data

The Illumina reads, generated from Maize seedlings transcriptome, were downloaded from NCBI Gene Expression Omnibus (ID: GSM448856) [40]. The reads, already trimmed for adaptors, were 7.922 millions in number.

The pre-processing of Illumina reads was done using MiroPipeline [11], which involved low complexity filtering, inclusion of reads without adaptor, and replacing identical sequences with single representatives. For mapping reads in miRDeep, the default choice of mapper, namely mega-blast [41], was replaced with one of faster mapper, namely SOAP-v2.2 [42]. Mapping was done onto unmasked Maize genome [43], with repeated hits allowed, and the maximum number of mismatches was set to 1. The hits were later filtered for number of multiple best hits, a maximum of 20 was set as a threshold (so that reads repetitive in nature get excluded, at the same time those most likely mapping to multiple members of a miRNA gene family are considered), and then converted into miRDeep compatible format. The hits with protein coding sequences and with various types of non-coding RNA (eg., rRNA, tRNA) were also filtered out [44,45]. The excision length was set to a maximum of 300. In order to speed up the mapping of filtered reads onto precursors (default program: auto_blast.pl of miRDeep package), we used MiroPipeline (configured for using seqmap as an alignment tool) [11]. The core program (*miRDeep.pl*) was run with score threshold 0 and stability check on. One of the miRDeep's filtering criteria, to exclude candidates with bifurcation in secondary structure, was set off to improve sensitivity.

The key syntactic changes for incorporating plant specific parameters in PERL scripts of miRDeep are listed in Additional file 5.

## Conclusions

The difference in properties of plant and animal miRNAs has large impact on the statistical features used for miRNA prediction. The parameters used for animal miRNA prediction cannot be used to predict plant miRNAs. Among the statistical features, the minimum free energy was found to have marginal difference between real and background in monocots. However dicots showed a different behavior wherein MFE scoring is potentially a key discriminator. The stability pattern in plant miRNAs was different to animals, in particular among the background sequences. The positional conservation profile was relatively longer in plants, so does the associated frequencies. The new set of parameters identified in this study will substantially improve our capacity to predict plant miRNAs.

## Conflict of interest

The authors declare that they do not have competing interests.

## Authors' contributions

VT, SW, MX, RB, WPQ, AM, and XZ conceived the study. VT and SW carried out the experiments. VT, SW, AM, XZ analyzed the results and wrote the paper. All authors read and approved the final manuscript.

## Supplementary Material

Additional file 1 **Figure 1**. Mean MFE as a function of (precursor) length. The vertical bars display the standard deviation. The best fit linear curve has a slope of 0.48. Click here for file

Additional file 2 **Figure 1**. Comparison of cumulative frequency distributions of (length normalized) MFE of real and background precursors from dicot species. These precursors are of length 260-290 nt. Bgr: background. Click here for file

Additional file 3 **Figure 1**. Best fit curve of Gumbel distribution (minimum) for the cumulative distributions of MFE of real and background precursors (length: 260-290 nt). Except for small range of normalized MFE values, largely in middle, the corresponding curves do not fit well. Click here for file

Additional file 4 **Figure 1**. Cumulative frequency distribution of *p-value *of background precursors of five species (size = 300 nt). Those from dicot species have higher stability, this, rendering them less distinguishable from the real precursors. Click here for file

Additional file 5 **Text**. Key changes made in the syntax of miRDeep to incorporate plant-specific parameters. Click here for file
