# Supplementary material for: Characterization of statistical features for plant microRNA prediction
Source: BMC Genomics. 2011 Feb 16;12:108. doi: 10.1186/1471-2164-12-108 (PMC3053258; doi:10.1186/1471-2164-12-108)
Supplement: Additional file 5 — Text. Key changes made in the syntax of miRDeep to incorporate plant-specific parameters. [file 1471-2164-12-108-S5.DOC]

Notes:

1. The changes specified here are in two of the PERL scripts of miRDeep, downloaded on April 2010.
2. The syntax to be changed has been referred by line numbers. Changes should preferably be made in decreasing order of line numbers.
3. For convenience, both the syntax to be replaced (labelled with '<') and its replacement (labelled with '>'), have been explicitly mentioned. Make sure to REMOVE THERE LABELS from the PERL scripts.

===========================================

**PROGRAM NAME: excise_candidate.pl**

===========================================

**TOTAL 2 CHANGES:**

===========================================

1. **REPLACE lines #174-182:**

**Explanation: To ignore the case where overlapping size is over 30 nt in length, and also increase the excision length**

< if(($subject_end-$subject_beg)>30){

< excise_position(\$subject,\$seq,\$seq_lng,\$strand,\($subject_beg-22),\($subject_end+22),\$count);

< $count++;

< }else{

< excise_position(\$subject,\$seq,\$seq_lng,\$strand,\($subject_beg-22),\($subject_beg+87),\$count);

< excise_position(\$subject,\$seq,\$seq_lng,\$strand,\($subject_end-87),\($subject_end+22),\$count);

< }

**-------By-------**

> # if(($subject_end-$subject_beg)>30){

> # excise_position(\$subject,\$seq,\$seq_lng,\$strand,\($subject_beg-22),\($subject_end+22),\$count);

> # $count++;

> # }else{

> excise_position(\$subject,\$seq,\$seq_lng,\$strand,\($subject_beg-22),\($subject_beg+276),\$count);

> excise_position(\$subject,\$seq,\$seq_lng,\$strand,\($subject_end-276),\($subject_end+22),\$count);

> # }

______________________________________________________

2. **REPLACE line #201:**

**Explanation: To adjust the maximum excision length**

< if(140<$excise_lng){return;}

**-------By-------**

> if(300<$excise_lng){return;}

____________________________________________________

===========================================

**PROGRAM NAME: miRDeep.pl**

===========================================

**TOTAL 6 CHANGES**

===========================================

1. **REPLACE lines #1451-1461:**

**Explanation: Since the MFE scoring here uses log-odds, so the subroutine score_mfe() needs to be modified**

< my $mfe=shift;

< #numerical value, minimum 1

< my $mfe_adj=max2(1,-$mfe);

< #parameters of known precursors and background hairpins, scale and location

< my $prob_test=prob_gumbel_discretized($mfe_adj,5.5,32);

< my $prob_background=prob_gumbel_discretized($mfe_adj,4.8,23);

< my $odds=$prob_test/$prob_background;

< my $log_odds=log($odds);

**-------By-------**

> my ($mfe,$pri_lng)=@_;

> #normalize the MFE by length

> my $mfe_adj=$mfe/$pri_lng;

> #instead of finding individual functions for real and bgr, the one for log-odds was directly obtained.

> #Its a sigmoid func. with +ve x. f(x)=a/(b+exp(x*c))

> my $param_a=1.339e-12; my $param_b= 2.77826e-13; my $param_c= 45.8426; my $log_odds=0;

> $log_odds=$param_a/($param_b+exp($mfe_adj*$param_c));

____________________________________________________

2. **COMMENT lines #1468-1496 (Put '#' in the beginning of specified lines):**

**Explanation: Two subroutines, prob_gumbel_discretized() and cdf_gumbel(), are no longer required**

______________________________________________________

3. **REPLACE #515-521:**

**Explanation: Since duplex properties are different so modify/add filters of subroutine 'pass_filterining_structure()'**

< unless(no_bifurcations_precursor()){$ret=0;}

< #minimum 14 base pairings in duplex

< unless(bp_duplex()>=14){$ret=0; filter_s("too few pairings in duplex");}

< #not more than 6 nt difference between mature and star length

< unless(-6<diff_lng() and diff_lng()<6){$ret=0; filter_s("too big difference between mature and star length") }

**-------By-------**

> # unless(no_bifurcations_precursor()){$ret=0;}

> #minimum 15 (default=14) base pairings in duplex

> unless(bp_duplex()>=15){$ret=0; filter_s("too few pairings in duplex");}

> #not more than 3 (defualt=5) nt difference between mature and star length

> unless(-3<=diff_lng() and diff_lng()<=3) {$ret=0; filter_s("too large difference between mature and star length"); }

> #maximum 5 unpaired bases

> unless (ttl_unpaired()<6) {$ret=0; filter_s("too many unpaired bases in duplex");}

> #maximum 3 consecutive unpaired bases

> unless (consc_unpaired()<=3) {$ret=0; filter_s("too long size of consecutive unpaired bases in duplex");}

____________________________________________________

4. **ADD TWO SUBROUTINES TO THE END:**

**Explanation: Since filters were added to pass_filterining_structure(), new subroutines are now required- i) ttl_unpaired()', ii) 'consc_unpaired()'**

> sub ttl_unpaired{

> my $mature_struct=$hash_comp{"mature_struct"};

> #total unpaired

> my $count_unpaired=$mature_struct=~s/\./\./g;

> return $count_unpaired;

> }

> sub consc_unpaired{

> my $mature_struct=$hash_comp{"mature_struct"};

> #replace first the paired by '1', and split the string based on '1'

> $mature_struct=~s/\(+/1/g; $mature_struct=~s/\)+/1/g;

> my @unprd=split '1',$mature_struct;

> my @list_unprd="";

> shift @list_unprd;

> #count them and store in an array

> my $elem=""; my $num_unprd="";

> foreach $elem(@unprd) {

> if ($elem=~/\./g) {$num_unprd=$elem=~s/\./\./g;push @list_unprd,$num_unprd;}

> }

> #if the array is not NULL, then SORT the consc_unprd in ASCENDING order

> if ($#list_unprd>=0){

> @list_unprd= (sort {$a<=>$b} @list_unprd);

> return pop(@list_unprd);

> }

> else {return 0;}

> }

______________________________________________________

5. **REPLACE line #256:**

**Explanation: One aditional argument passed in subroutine 'score_mfe()'**

< my $score_mfe=score_mfe($hash_comp{"pri_mfe"});

**-------By-------**

> my $score_mfe=score_mfe($hash_comp{"pri_mfe"},$hash_comp{"pri_end"});

_____________________________________________________

6. **REPLACE lines #70-77:**

**Explanation: Finally provide new parameters**

< my $nucleus_lng=7;

< my $score_star=3.9;

< my $score_star_not=-1.3;

< my $score_nucleus=3;

< my $score_nucleus_not=-0.6;

< my $score_randfold=1.6;

< my $score_randfold_not=-2.2;

**-------By-------**

> my $nucleus_lng=11; #new=11 (2..12) Default=7

> my $score_star=3.9;

> my $score_star_not=-1.3;

> my $score_nucleus=7.635; #new... 7.635 (1 hit per 3000 bgr) or 8.146 (1 hit per 5000 bgr) Default=3

> my $score_nucleus_not=-1.17; #new...-1.17 Default=-0.6

> my $score_randfold=1.37; #new... 1.37 Default=1.6

> my $score_randfold_not=-3.624; #new...-3.624 Default=-2.2

===========================================

**ADDITIONAL CHANGES IN THE ABOVE FOR DICOTS**

===========================================

**TOTAL 2 CHANGES**

===========================================

1. **Replace parameters in score_mfe()**

< my $param_a=1.339e-12; my $param_b= 2.77826e-13; my $param_c= 45.8426; my $log_odds=0;

**-------By-------**

> my $param_a=4.46e-4; my $param_b= 9.125e-5; my $param_c= 26.9293; my $log_odds=0;

_______________________________________________________

2. **REPLACE lines #76-77: global parameters to be modified**

< my $score_randfold=1.37; #new... 1.37 Default=1.6

< my $score_randfold_not=-3.624; #new...-3.624 Default=-2.2

**-------By-------**

> my $score_randfold=0.631; #new... monocot=1.37 Default=1.6

> my $score_randfold_not=-3.1749; #new...monocot=-3.624 Default=-2.2
